# Pharyngocutaneous Fistula as a Rare Late Postoperative Complication Following Submandibulectomy: A Case Report

**Published:** 2017-07

**Authors:** Leila Mashali, Somayeh Araghi

**Affiliations:** 1 *Department of Otolaryngology Head and Neck Surgery, Imam Khomeini Hospital, Ahvaz Jundishapur University of Medical Sciences, Ahvaz, Iran.*; 2 *Department of Otolaryngology Head and Neck Surgery, Tehran University of Medical Sciences, Tehran , Iran.*

**Keywords:** Pharyngocutaneous fistula, Postoperative complication, Submandibular gland excision, Submandibulectomy

## Abstract

**Introduction:**

Submandibular gland excision is the gold standard treatment for submandibular gland disease. Although submandibulectomy is a relatively standardized surgical procedure, complications are frequently reported. These complications include nerve paralysis or paresis, aesthetic sequelae, hematoma, salivary fistulas or sialoceles, wound infections, hypertrophic scars and inflammations caused by residual lithiasis in the salivary duct.

**Case Report:**

We report a case of a rare complication of submandibular gland excision, pharyngocutaneous fistula, which appeared 6 years after previous surgery. The patient underwent surgery, during which a fistula tract from the skin to the pharynx was found and excised.

**Conclusion:**

The authors believe that inappropriate execution of the surgical procedure could result in postoperative complications.

## Introduction

The submandibular gland is the second largest salivary gland in the human body. Each submandibular gland weighs approximately 10–15 g, and is anatomically divided into the superficial and deep parts; each separated by the mylohyoid muscle. Facial vessels and three important nerves including the hypoglossal, lingual nerve, and marginal mandibular branch of the facial nerve proceed in the medial part of the submandibular gland. The submandibular gland produces 71% of daily saliva, and the secretion is composed of serous and mucoid components. Sialolithiasis is the most common salivary disease and the most common cause of salivary gland dysfunction. Eighty percent of salivary stones are seen in the submandibular gland (1). Submandibular gland surgery is performed by many different surgical specialties for a variety of indications; the commonest of which is chronic sialadenitis with or without calculus formation (2). Transcervical extirpation of the gland is the gold standard of treatment for submandibular gland disease (3). Although submaxillectomy is a relatively standardized surgical procedure, complications are still frequently reported by different groups. These complications include nerve paralysis or paresis, aesthetic sequelae, hematoma, salivary fistulas or sialoceles, wound infections, hypertrophic scars, and inflammations caused by residual lithiasis in the salivary duct (4).

## Case Report

A 23-year-old male patient was admitted to our hospital with left submandibular discharge from a fistula which first presented 1 year previously and which occurred on eating. He had a scar due to the incision of a previous transcervical submandibulectomy (external approach) because of sialolithiasis about 7 years ago. 

**Fig 1:**
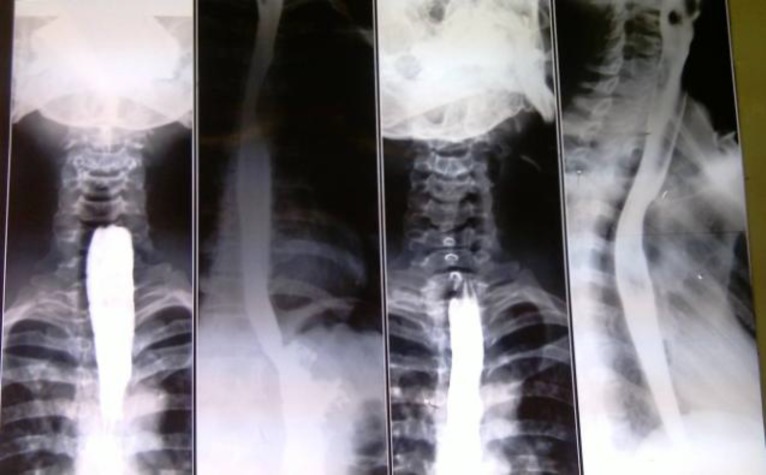
Esophagography

A complete history and physical examination revealed no additional family or other important information (cervical planes manipulation due to previous surgery). The patient’s esophagography revealed a fistula tract in the upper third of the esophagus but no other pathologic findings (Fig.1). On undergoing surgery, a fistula tract from the skin to the pharynx was found and excised.

**Fig 2:**
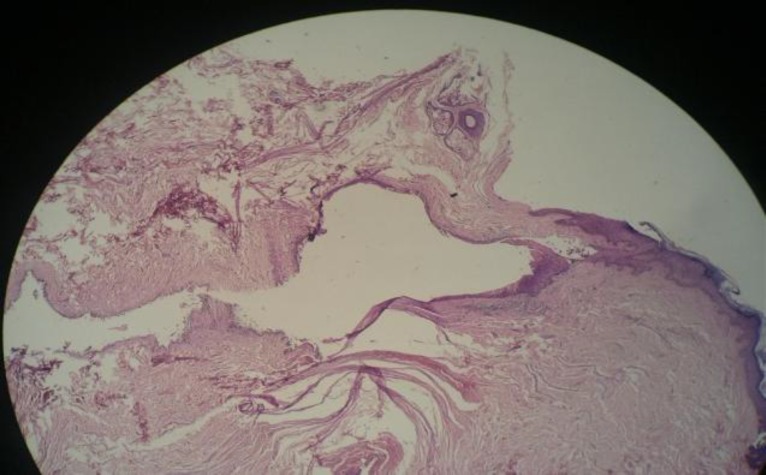
Pathology slides

 Pathology of the specimen macroscopically showed a piece of brownish colored tissue (1.5×1×1.5 cm in size). Microscopically, sections revealed skin tissue with the dermal tract lined by bland squamous epithelium, compatible with a clinical diagnosis of pharyngocutaneous fistula (Fig.2).

## Discussion

Preuss et al. reported a number of perioperative complications after submandibulectomy, including hematoma, wound infection, saliva fistula, transient palsy of lingual nerve, and transient palsy of the mandibular branch (5).

In another study, Hernando et al. reported complications subsequent to submaxillectomy frequently published by different groups, including nerve paralysis or paresis, aesthetic sequelae, hematoma, salivary fistulas or sialoceles, wound infections, hypertrophic scars and inflammations caused by residual lithiasis in the salivary duct. In their series, cases of marginal paresis were rare, with their main complication being postoperative infections in cases of chronic sialadenitis (4).

In a study by Milton et al., the following complications after submandibular gland excision were reported: wound infection (9%) all of which were resolved within weeks; hematoma (10%) requiring two patients to return to the اhospital for transfusion; recurrent disease (2%) in which stones were found in the duct at follow-up requiring further surgery; miscellaneous (2%), including (i) neuroma related to silk suture and (ii) chorda tympani syndrome; sinus (3%) in which discharge continued for several weeks or months (no surgery was required); scarring (4%) that was not considered significant (2). Other studies reported similar complications following submandibular gland excision (1,3). We present pharyngocutaneous fistula as a rare late complication of the submandibulectomy, which to the best of our knowledge has not previously been reported in the literature.

 In a case report, Abu-Ella presented a submandibular gossypiboma mimicking a salivary fistula. This was the case of a 27-year-old man who suffered a persistent discharging sinus for 8 years following excision of a right submandibular gland. Computed tomography fistulography was performed showing a blind track ending in the cavity just beneath the floor of the mouth. Neck exploration eventually revealed two gauze swabs that were tightly packed in the area of the submandibular duct (6). This case report shows a rare and late postoperative complication following subman- dibulectomy due to inappropriate execution of the surgical procedure and distortion of the cervical planes. Such cases are rarely reported.

## Conclusion

The authors believe that inappropriate execution of the surgical procedure could result in postoperative complications following submandibulectomy.
